# Five-Year Field Results and Long-Term Effectiveness of 20 mg/kg Liposomal Amphotericin B (Ambisome) for Visceral Leishmaniasis in Bihar, India

**DOI:** 10.1371/journal.pntd.0002603

**Published:** 2014-01-02

**Authors:** Sakib Burza, Prabhat K. Sinha, Raman Mahajan, María Angeles Lima, Gaurab Mitra, Neena Verma, Manica Balasegarem, Pradeep Das

**Affiliations:** 1 Médecins Sans Frontières, New Delhi, India; 2 Rajendra Memorial Research Institute of Medical Sciences, Patna, Bihar, India; 3 Médecins Sans Frontières, Barcelona, Spain; 4 ACCESS Campaign, Geneva, Switzerland; Emory University, United States of America

## Abstract

**Background:**

Visceral Leishmaniasis (VL; also known as Kala-azar) is an ultimately fatal disease endemic in Bihar. A 2007 observational cohort study in Bihar of 251 patients with VL treated with 20 mg/Kg intravenous liposomal amphotericin B (Ambisome) demonstrated a 98% cure rate at 6-months. Between July 2007 and August 2012, Médecins Sans Frontières (MSF) and the Rajendra Memorial Research Institute (RMRI) implemented a VL treatment project in Bihar, India—an area highly endemic for *Leishmania donovani*—using this regimen as first-line treatment.

**Methods and Principal Findings:**

Intravenous Ambisome 20 mg/kg was administered in four doses of 5 mg/kg over 4–10 days, depending on the severity of disease. Initial clinical cure at discharge was defined as improved symptoms, cessation of fever, and recession of spleen enlargement. This observational retrospective cohort study describes 8749 patients with laboratory-confirmed primary VL treated over a 5-year period: 1396 at primary healthcare centers, 7189 at hospital, and 164 at treatment camps. Initial clinical cure was achieved in 99.3% of patients (8692/8749); 0.3% of patients (26/8749) defaulted from treatment and 0.4% (31/8749) died. Overall, 1.8% of patients (161/8749) were co-infected with HIV and 0.6% (51/8749) with tuberculosis. Treatment was discontinued because of severe allergic reactions in 0.1% of patients (7/8749). Overall, 27 patients (0.3%) were readmitted with post Kala-azar dermal leishmaniasis (PKDL). Risk factors for late presentation included female sex, age >15 years and being from a scheduled caste.

In 2012, a long-term efficacy survey in the same area of Bihar determined relapse rates of VL after 5 years' intervention with Ambisome. Of 984 immunocompetent patients discharged between September 2010 and December 2011, 827 (84.0%) were traced in order to determine their long-term outcomes. Of these, 20 patients (2.4%) had relapsed or received further treatment for VL. Of those completing 6, 12, and 15 month follow-up, 0.3% (2/767), 3.7% (14/383), and 2.4% (4/164), respectively, had relapsed. The mean ±SD time-to-relapse was 9.6±3.0 months.

**Significance:**

This is the largest cohort of VL patients treated with 20 mg/kg Ambisome worldwide. The drug has high initial and long-term efficacy, and a low rate of adverse reactions when administered under field conditions in Bihar, India. Although challenging, its use as first line treatment in rural settings in Bihar is safe and feasible.

## Author Summary

Visceral leishmaniasis (VL), also known as Kala-azar, is a protozoan parasitic disease transmitted by phlebotomine sandflies. After malaria, VL accounts for the second-highest burden of parasitic diseases worldwide. India has half of all the VL patients worldwide, of which up to 90% are in Bihar state. Between 2007–2012, Médecins Sans Frontières (MSF) together with the Rajendra Memorial Research Institute (RMRI) supported existing health structures in Vaishali district, Bihar, by treating 8749 VL patients with intravenous liposomal amphotericin B (Ambisome) 20 mg/kg. Initial cure rate was high, with low default and mortality rates. We describe the demographic and clinical characteristics of this large patient group, including risk factors for late presentation. Ambisome can be given in short courses and is safer, better tolerated, and more effective than existing VL treatments. The MSF-supported program treated nearly 1400 ambulatory patients in rural primary healthcare centers, using existing government facilities and non-incentivized staff, demonstrating that a resource-limited healthcare system in a rural setting can meet the cold chain and human resource standards required to administer Ambisome safely. We also describe a long-term follow-up survey showing a continuing low VL relapse rate after 5 years of routine use of Ambisome. More relapses occurred after 6-month follow-up; suggesting a 12-month follow-up in future VL studies may be more appropriate.

## Introduction

Visceral leishmaniasis (VL), also known as Kala-azar, is a protozoan parasitic disease transmitted by phlebotomine sandflies. It is estimated that in 2004 a total of 1,071,743 disability-adjusted life years were lost to VL in South East Asia alone [1]; if left untreated the disease is fatal. India contributes the highest number of VL cases worldwide and in 2011 reported 33,187 cases, of which 76% originated in Bihar state [2]., Despite a fall in reported cases in 2012 in India, there is evidence of substantial under-reporting of VL [3], [4] and the estimated annual incidence lies between 146,700–282,800 cases [5]. In 2005, India, Nepal, and Bangladesh signed a tripartite memorandum of understanding committing to the elimination of VL by 2015.

In 2007, Médecins Sans Frontières (MSF), in collaboration with the Rajendra Memorial Research Institute of Medical Science (RMRI; Patna, Bihar, India), and the National Vector Borne Disease Control Programme of India, carried out an observational cohort study in Vaishali district, Bihar of 251 VL patients treated using 20 mg/kg liposomal amphotericin B (Ambisome; Gilead Pharmaceuticals, Foster City, CA, USA) as first-line treatment. All patients were admitted to the district hospital for the duration of treatment, and given four doses of Ambisome 5 mg/kg over a 10-day period. The intent-to-treat analysis yielded a treatment effectiveness rate of 98.8% at 6 months, with no relapses and an excellent safety profile [6]. In coordination with the RMRI, MSF shortly thereafter signed an agreement with the State Health Society, Bihar to reinforce the existing programme by identifying VL patients in Vaishali district and treating with Ambisome 20 mg/kg.

Ambisome is a brand name for Liposomal Amphotericin B. There are a number of preparations of Liposomal Amphotericin B available on the market; however due to the lack of standard and widely applicable regulations or guidance for liposomal technology, it is important that this specific preparation be named. At time of publication, none of the rival preparations have undergone peer reviewed non-inferiority studies against Ambisome nor received stringent regulatory approval for use in VL. It is for this reason that MSF and the WHO currently only use Ambisome rather than other preparations. However it is urgent that clear regulatory guidelines for endemic countries be established by a normative setting organisation like the WHO and other existing formulations be formally evaluated [7].

This observational retrospective cohort study describes the field outcomes and experiences of the programme over the subsequent 5 years, discussing the challenges of implementing Ambisome treatment at the field level, and describes the long-term outcomes of treatment after 5 years of routine operational use.

## Methods

MSF developed an integrated programme within the existing healthcare facilities of Vaishali district that utilised the district hospital for inpatient care and five rural primary healthcare centers (PHCs) for ambulatory treatment in the community. Vaishali district, with a population of 3.5 million, lies at the center of Bihar's VL belt, and is surrounded by three of the highest endemic districts for VL in the state. A comprehensive strategy of information, education, and communication (IEC) was implemented within five administrative blocks within the district where MSF supported government facilities. The intention was to treat patients with complicated VL as inpatients at the district hospital, and other VL patients in the PHCs, however the widespread lack of community belief in the PHC system [8] meant that it was understood from the outset that the majority of patients who could have been treated in the PHCs would seek care directly at the district hospital. Patients diagnosed with VL at PHC level aged <2 years or >55 years, pregnant, lactating, severely malnourished, had a hemoglobin (Hb) level of <5 g/dL, gave a history of a previous episode of VL that had been treated and resulted in apparent cure, or were known to be infected with HIV were referred to the district hospital for treatment.

MSF provided all human resource requirements for the dedicated VL treatment ward at the district hospital. However, at the PHCs, the treatment programme relied on existing Ministry of Health staff who were trained and mentored by MSF, but who received no financial incentive. Diagnostic kits and drugs were supplied and monitored by MSF. MSF initially managed the Ambisome cold chain through a logistically complex and unreliable passive cool box/ice pack mechanism. However, a simpler solution was implemented whereby the State Health Society, Bihar provided ice-lined refrigerators (ILRs) specifically for Ambisome at each treatment site. These ILRs require only 8 hours per day of constant electricity to maintain a temperature of 2–8°C for 24 hours, and are routinely used in India's Extended Programme of Immunisation [9].

### Patients

All patients with a history consistent with VL (fever of >2 weeks duration and splenomegaly) were confirmed using rK39 rapid diagnostic tests (DiaMed-IT LEISH; DiaMed AG, Cressier, Switzerland). Patients presenting with relapse, or in whom there was continued suspicion of VL despite negative diagnostic tests, were referred to a tertiary center (RMRI) for parasitological confirmation through splenic or bone marrow aspiration. The RMRI is a tertiary research institution that specializes in all aspects of VL research and treatment.

General demographic data were recorded for all patients diagnosed with VL, in addition to clinical history, Hb level, height, weight and malaria rapid diagnostic test result. Also recorded was ‘caste’, a form of social stratification used in India, and the categories used in the study were: scheduled caste, other backward class, scheduled tribe, and general category. Other backward class is a collective term used by the government of India for castes that are educationally and socially disadvantaged. Scheduled caste and scheduled tribe are terms used for two groups of historically disadvantaged people recognized in the Constitution of India. These three groups combined account for approximately 60% of India's population. General category comprises those who do not fit within the other categories and are not considered to be disadvantaged.

Initially only inpatients deemed to be at high risk of HIV (e.g. those experiencing a relapse of VL or with a history suggestive of higher risk, such as migrant workers) were offered an HIV test, however this policy was changed in March 2011 so all patients treated at the district hospital level aged >14 years were offered testing. All women ≥14 were offered a pregnancy test.

### Treatment regimen

Patients received four doses of 5 mg/kg Ambisome over 4–10 days depending on the clinical severity of their illness. Initially all patients were treated on days 0, 1, 4, and 9. However, once the safety of the treatment was established, and because of increasing patient numbers and the hospital's limited capacity, the duration of treatment for all clinically stable inpatients was reduced to 4 consecutive days. The 10-day regimen was maintained for severely ill inpatients and for ambulatory patients treated at the PHCs.

Initial cure was defined as improvement of symptoms, cessation of fever, and reduction of spleen size at time of discharge. Considering the risks of splenic puncture and in light of a previous study showing >98% cure rate at 6 months using the same regimen [6], test-of-cure was planned only on those patients with suspicion of treatment failure, of whom there were none. Patients received health education regarding VL, and advice given to return or be actively contacted at 3, 6, and 12 months for follow-up. Additionally, all patients received health education regarding post Kala-azar dermal leishmaniasis (PKDL) and the possibility of relapse of VL, and were advised to return to the district hospital if either situation occurred.

### Follow-up

Towards the end of the 5-year period of analysis, an active follow-up survey was conducted to determine the long-term relapse rates for a sub-cohort of VL patients. All patients not known to be HIV-positive residing within 8 of the 16 administrative blocks constituting Vaishali district, and who had completed VL treatment between two reference dates (September 2010 to December 2011) spanning 18 months prior to the survey date (March 2012) were traced. Any history of mortality, relapse, or retreatment was recorded. The eight administrative blocks, with an average of 215,000 residents per block, were selected for ease of access and highest density of patients treated within the programme.

### Statistical analysis

All data were entered into a standard Microsoft Excel database; double data-entry was not done. Regular database cleaning comprised checks for inconsistencies with reference to source documents where necessary. An epidemiologist ensured the database was well maintained and regularly audited the quality of data transfer. Nutritional status (Body Mass Index) was assessed using weight and height data, whilst World Health Organization Anthro and Anthro Plus software (Geneva, Switzerland) was used to calculate a weight-for-height Z-score for children aged <5 years and a BMI-for-age Z-score for those aged ≥5–19 years.

A retrospective analysis of all routinely collected program data was conducted using SPSS version 19 statistical software (IBM, Chicago, IL, USA). A multivariate logistic regression model was also developed to determine risk factors significantly (p<0.05) associated with being a ‘late presenter’ on univariate analysis (i.e. >4 weeks of illness prior to treatment).

### Ethics statement

This analysis met the Médecins Sans Frontières Institutional Ethics Review Committee criteria for a study involving the analysis of routinely collected program data. Although a new treatment in the Indian setting, the programme utilised a recognised treatment for VL and was run in coordination with the State Health Society through a memorandum of understanding, which is the usual procedure for NGOs operating in this context. All electronic data were analysed anonymously.

## Results

**Figure 1:**
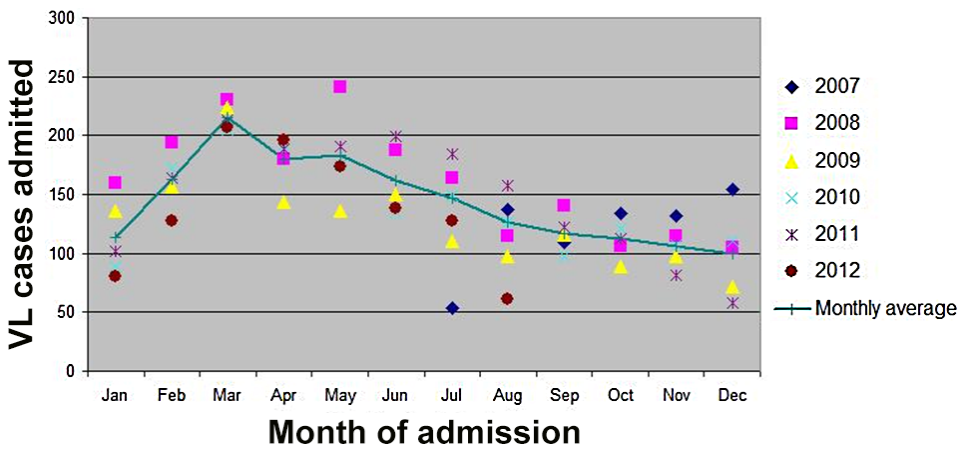
Admissions by month between 2007 and 2012.

A total of 8749 patients were treated for VL during the 5-year MSF-supported program (July 2007 and August 2012), with admissions following a similar seasonal pattern each year (Figure 1).

### Patient characteristics

**Figure 2:**
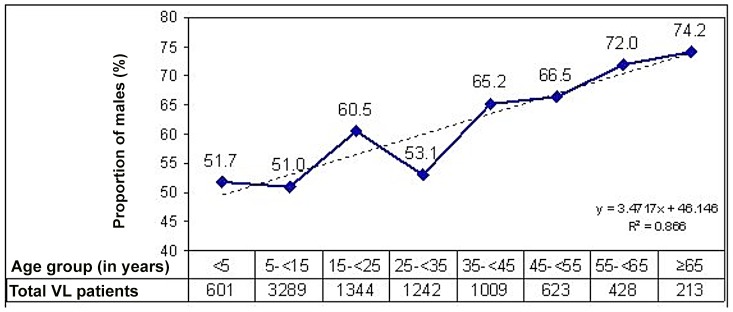
Age/sex ratio graph showing proportion of males admitted into programme by age.

Of the 8749 patients, 42.9% of patients were female, a proportion substantially lower than the 47.12% of females in the background population sex distribution [10]. The mean ±SD age was 22.7±17.1 years (range 0.5–90); 44.5% of patients were aged <15 years, of whom 6.9% were aged <5 years. As the age of patients increased, the proportion of males increased (Figure 2). The odds ratio (OR) of being male and enrolled into the program at age groups ≥15–34, ≥35–54, and ≥55 years versus baseline (<15 years) was 1.6 (95% CI 1.4–1.7), 2.0 (95% CI 1.8–2.2), and 2.5 (95% CI 2.1–3.1), respectively (p<0.01). Overall, age group accounted for 86% of the variability in sex ratio.

A total of 7338/8749 patients (83.8%) were from scheduled caste/tribe or other backward classes (Table 1). Of the cohort, 95.6% had primary VL and 4.4% gave a history of one or more previous episodes of VL. Of the latter, 4% presented with a history of a single episode for which they had received a course of treatment that had resulted in apparent recovery, whilst the remainder reported multiple episodes. Patients describing previous episodes of VL reported being treated with: amphotericin B deoxycholate (1.2%, n = 104), sodium stibogluconate (SSG; 1.5%, n = 135) or miltefosine (1.5%, n = 128) for their most recent episode of VL.

**Table 1:** Characteristics of the study population (N = 8749).

Variable		n (%)
Sex	Male	5000 (57.1)
	Female	3749 (42.9)
Age group, years	<5	601 (6.9)
	5 to <15	3289 (37.6)
	15 to <45	3595 (41.1)
	≥45	1264 (14.4)
Caste category	Scheduled caste	2524 (29.0)
	Other backward class	4809 (55.0)
	Scheduled tribe	5 (0.1)
	General Category	1361 (15.6)
	Missing	50 (0.6)
District of origin	Vaishali	6447 (73.7)
	Saran	1381 (15.8)
	Samastipur	430 (4.9)
	Muzaffarpur	237 (2.7)
	Other	254 (2.9)

A total of 1813 patients with VL were registered at the PHC, of whom 416 (22.9%) appeared to meet the referral criteria and were referred to the district hospital for treatment. Therefore, 16.0% (1397/8749) of patients were treated at rural primary health centers, 78.6% (6874/8749) were treated at the district hospital, and 3.6% (314/8749) were treated in the tertiary referral center (RMRI). The remaining 1.9% (164/8749) of patients were treated in the community by MSF during occasional mobile treatment campaigns during the 5-year period. Overall, 73.7% of patients originated from within Vaishali district; however, this proportion decreased from 81.3% in 2008 to 65.7% in 2011, reflecting an increasing number seeking care in the programme from outside the district. The proportion of patients residing in one of the blocks containing a MSF supported PHC whose first presentation to the programme was at the level of the PHC (as opposed to the district hospital) remained similar at 34.9%, 40.7%, 33.5% and 32.2% for the whole years 2008–2011 respectively. The Ambisome 20 mg/kg total dose was received by 79.2% (6928/8749) of patients over 7–10 days and 20.2% (1767/8749) of patients over 4 consecutive days.

### Clinical characteristics

The clinical characteristics of all the patients are shown in Table 2. Of note, the mean Hb level at admission was 8.4 g/dL, with 46.1% of patients (4034/8749) presenting with a Hb level of <8 g/dL. The mean spleen size (palpable below the costal margin) at admission was 6.1 cm; 34.5% of the cohort presented with a spleen size >6 cm. Both these indicators directly correlated with duration of illness prior to treatment (Pearsons Correlation r = 0.215 and r = −0.108 for Hb and Spleen size respectively, p<0.001). Compared with early presenters, late presenters had slightly lower mean Hb levels (8.1 vs 8.6 g/dL) and greater splenomegaly (6.9 vs 5.5 cm). Although no association between different age groups and spleen size at the time of admission was seen, there was a direct correlation between increasing age and increasing Hb (Pearsons Correlation r = 0.232, p≤0.001).

**Table 2:** Clinical characteristics of treated patients with visceral leishmaniasis (N = 8749).

Variable*		n (%)	Mean ±SD (range)
Diagnostic criteria [ = 8749]**	rK39 positive	8730 (99.8)	–
	Parasitological confirmation	656 (7.5)	–
Illness duration before treatment, weeks [ = 8741]	<4	5085 (58.1)	–
	4–8	2153 (24.6)	–
	>8	1503 (17.2)	–
			6.4±6.1 (0–159)
Spleen size, cm [ = 8744]	<3	1272 (14.5)	–
	3–6	4453 (50.9)	–
	>6	3019 (34.5)	–
			6.1±3.8 (0–32)
Hemoglobin, g/dL [ = 8723]	<6	1134 (13.0)	–
	6–8	2900 (33.1)	–
	>8	4689 (53.6)	–
			8.4±2.2 (2–18)
History of relapse [ = 8741]	No relapse	8364 (95.6)	–
	Single relapse	352 (4.0)	–
	Multiple relapses	33 (0.4)	–
History of previous treatment for VL ( = 8741)	No treatment	8364 (95.6)	–
	Sodium stilbogluconate (SSG)	135 (1.5)	–
	Amphotericin B deoxycholate	104 (1.2)	–
	Miltefosine	128 (1.5)	–
	Other	18 (0.2)	–
Nutritional status ( = 7254)***	Normal	4292 (59.2)	–
	Moderate acute malnutrition	1656 (22.8)	–
	Severe acute malnutrition	1306 (18.0)	–
Co-infections	HIV	161 (1.8)	–
	Tuberculosis	51 (0.6)	–
	Malaria	11 (0.1)	–
Pregnant		49 (0.6)	–

Where n<8749, data is missing.

Patients may have been diagnosed by rK39 and/or parasite confirmation.

Includes HIV +ve patients.

### Pregnancy and patients with HIV

Of the 3749 female patients within the cohort, 1810 were aged <14 year and were not offered pregnancy tests. Women with a documented history of hysterectomy or sterilization (a common and encouraged form of long-term contraception in India) were also not offered tests. Thus of the 1939 females aged ≥14 years, 1783 (92%) had tests performed of which 49 (2.75%) were positive at the time of treatment. This represented 1.3% of all women within the cohort (n = 3749). Of the pregnant patients, the mean ±SD age was 25.4±5.6 years, and the mean ±SD Hb level at admission was 7.6±1.8 g/dL. The mean ±SD duration of illness of pregnant patients was 6.6±5.6 weeks prior to treatment in the program.

Of the VL patients treated, 161 (1.8%) were HIV-positive (Table 3). Of these, 26 had been previously diagnosed with HIV and already on antiretroviral therapy. The odds of being HIV-positive and having previously experienced a single or multiple episodes of VL at time of admission was 13.6-times higher than in the overall cohort (95% CI 9.7–19.0; p<0.001).

**Table 3:** Characteristics of patients with Visceral Leishmaniasis and HIV co-infection (N = 161).

Variable		n (%)	Mean ±SD (range)
Gender	Male	134 (83.2)	
	Female	27 (16.8)	
Age group, years	<5	0 (0)	
	5 to <15	5 (3.1)	
	15 to <45	119 (73.9)	
	≥45	37 (23.0)	
			36.5±10.4 (7–70)
Nutritional status	Normal	90 (55.9)	
	Moderate acute malnutrition	33 (20.5)	
	Severe acute malnutrition	36 (22.4)	
	Missing	2 (1.2)	
Previous VL treatment	None	99 (61.5)	
	Sodium stilbogluconate (SSG)	15 (9.3)	
	Amphotericin B deoxycholate	24 (14.9)	
	Miltefosine	19 (11.8)	
	Other	4 (2.5)	
Number of relapses	No history of relapse	99 (61.5)	
	Single relapse	48 (29.8)	
	Multiple relapse	14 (8.7)	
On ART at time of VL treatment	Yes	26 (16.1)	
	No	135 (83.9)	

ART, antiretroviral therapy; VL, visceral leishmaniasis.

### Nutritional status

Of the 7254 patients (including HIV positive), whose anthropometric data was recorded at baseline 40.8% (n = 2962) were malnourished, with 18.0% (n = 1306) having severe acute malnutrition and 22.8% (n = 1656) having moderate acute malnutrition. There was a higher prevalence of malnutrition in the younger age groups, with the odds (CI) of being malnourished amongst the <5 and 5 - ≤19 years age groups 1.7 (1.4–2.2) and 2.2 (2.0–2.4) times higher respectively compared to the >19 years age group (p<0.001). There was no significant difference between the global nutritional status of patients known to be HIV positive and the remainder of the cohort (43.4% vs 40.8% globally malnourished respectively, RR (95%CI) = 1.1 (0.9–1.3), p = 0.506), nor was there a significant difference in the prevalence of severe acute malnutrition (SAM) between patients known to be HIV positive and the remainder of the cohort – 22.6% vs 17.9% respectively, RR (95%CI) = 1.3 (0.95–1.7), p = 0.124.

### Initial cure rate

The rate of initial cure of VL treatment, defined as cessation of fever, improvement of symptoms and recession of spleen enlargement at the time of discharge, was achieved in 99.3% (8692/8749) of patients. A total of 26 (0.3%) patients defaulted after receiving ≥1 dose of Ambisome, and 31 (0.4%) died during treatment. The case fatality rate of HIV patients during treatment was 4/161 (2.5%), compared to 27/8588 (0.3%) for patients not known to be HIV positive. The relative risk of mortality during treatment in patients with HIV was 7.9 (95% CI 2.8–22.3) times higher than that of patients not known to be HIV positive (p<0.001).

### Late presentation

Over half of all patients (58.1%, n = 5085) reported feeling unwell for <4 weeks prior to receiving treatment in the program, whereas 24.6% and 17.2% of patients were unwell for 4–8 and >8 weeks, respectively. The median duration of illness prior to admission was 4 weeks (IQR 3–8), whilst the mean duration was 6.4 weeks (SD 6.1). The odds of late presentation (defined as presenting >4 weeks after developing symptoms) were significantly higher in females (OR 1.2; 95% CI 1.1–1.3; p = 0.001), those from a scheduled caste (OR 1.2; 95% CI 1.0–1.3; p = 0.03), and age ≥15 years (OR 1.4; 95% CI 1.3–1.6; p<0.001) (Table 4). Receiving treatment in the PHC setting (OR 0.6; 95% CI 0.6–0.7; p<0.001) and having had a previous episode of VL (OR 0.8; 95% CI 0.6–0.9; p = 0.013) appeared to have a negative association against late presentation. Patients being diagnosed at the PHC level reported a shorter duration of symptoms prior to receiving treatment (1.0 week less, CI 0.8–1.3, p<0.001) than those who presented directly to the hospital for diagnosis and treatment. Neither residing within one of the blocks that MSF supports nor receiving care in a mobile treatment camp affected time of presentation.

**Table 4:** Risk factors for late presentation with visceral leishmaniasis (N = 8741)^a^.

Risk factor		Total	Number of Late presenters	Risk of late presentation, %	Unadjusted odds ratio (95% CI)	P value	Adjusted odds ratio (95% CI)	p value
Sex	Female	3747	1641	43.8	1.2 (1.1–1.3)	0.001	1.2 (1.1–1.3)	<0.001
	Male	4994	2015	40.3	–		–	
Caste	Scheduled caste	2524	1144	45.3	1.2 (1.0–1.3)	0.03	1.2 (1.0–1.3)	0.02
	Other backward class	4805	1921	40.0	0.9 (0.8–1.1)	0.26	0.9 (0.8–1.1)	0.394
	Forward	1360	567	41.7	–		–	
Living in MSF-supported residential block	No	4496	1932	43	1.1 (1.0–1.2)	0.025	1.0 (0.9–1.1)	0.906
	Yes	4245	1724	40.6	–		–	
Previous relapse	Yes	382	136	35.6	0.8 (0.6–0.9)	0.013	0.7 (0.5–0.9)	0.001
	No	8359	3520	42.1	–		–	
Age	≥15 years	4852	2222	45.8	1.4 (1.3–1.6)	0.000	1.5 (1.3–1.6)	<0.001
	<15 years	3889	1434	36.9	–		–	
Treatment location	Treatment camp (ambulatory)	164	65	39.6	0.9 (0.6–1.2)	0.32	0.8 (0.6–1.1)	0.236
	Primary healthcare center (ambulatory)	1396	463	33.2	0.6 (0.6–0.7)	<0.001	0.6 (0.57–0.7)	<0.001
	Hospital (inpatient)	7181	3128	43.6	–		–	

^a^ Length of illness data missing in 8 patients.

### Safety

Of the total patients treated, 7.2% (628/8749) suffered adverse reactions during treatment with Ambisome; 0.1% (7/8749) patients stopped treatment because of severe allergic reactions. The most common recorded complaints were nausea/vomiting (3.1%), back pain (1.9%), urticaria (1.2%), and rigors (0.5%). Neither location nor duration of treatment were associated with significant differences in initial cure rate, default, or adverse events.

### Post Kala Azar Dermal Leishmaniasis (PKDL)

Twenty-seven (0.3%) VL patients returned passively to the program following treatment complaining of symptoms subsequently confirmed as PKDL. The mean ±SD lengths of time from completion of treatment to development of skin lesions (as reported by the patients) and completion of treatment to formal diagnosis of PKDL were 20.4±12.1 months (range, 5.4–44.8) and 27.4±11.7 months (range, 10.1–53.2), respectively.

### Follow-up

Passive follow-up rates within the program were low, with 53.2% (4653/8749), 38.1% (3334/8749), and 1.5% (129/8749) of patients presenting at 3, 6, and 12-month follow-up, respectively. As previously described, an active follow-up survey was conducted in March 2012 of all patients who completed VL treatment between September 2010 and December 2011, who were not known to be HIV-positive, and who resided in eight administrative blocks within Vaishali district. A total of 984 patients met the criteria and 84.0% (n = 827) were successfully traced. The 984 patients represent 45.7% of all admissions into the program during this time period. Apart from patient origin, there were no significant differences in demographic and clinical characteristics between this group and the overall study cohort at the time of admission into the program.

**Figure 3:**
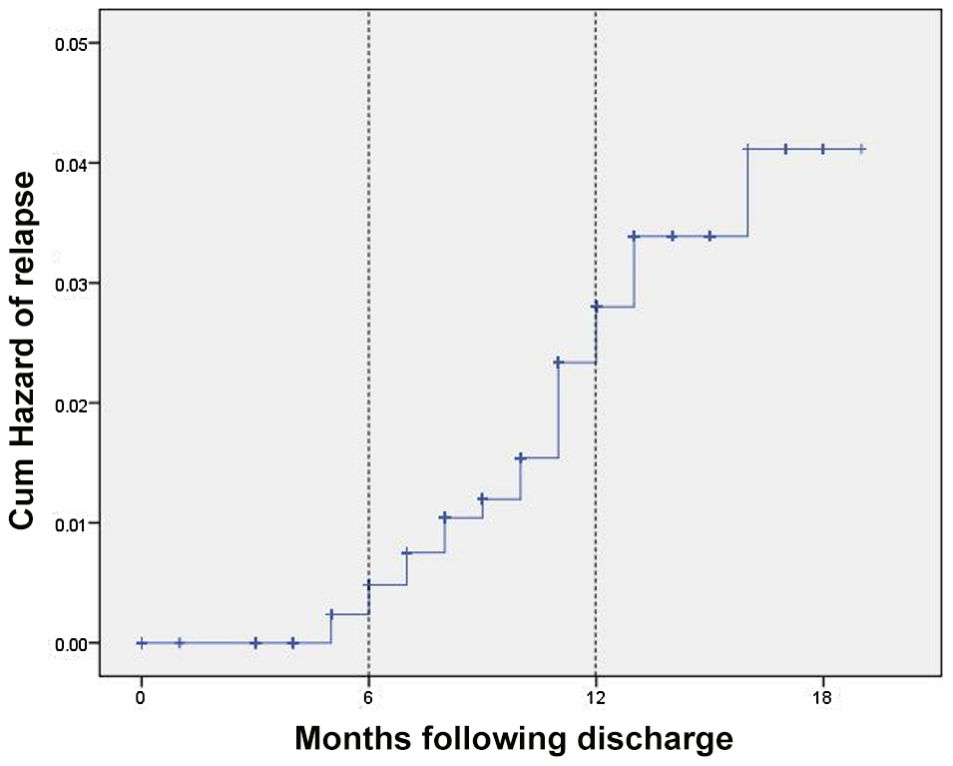
Censored Kaplan Meier curve showing the cumulative hazard of relapse over the time after discharge.

Table 5 details the outcomes of the active follow-up survey. Overall, 827, 767, 383, and 164 patients completed 3, 6, 12, and 15 months post-treatment, respectively (NOTE: these numbers are progressive and inclusive, e.g. those patients completing 12-month follow-up who had not relapsed were included in the denominator of the 6-month follow-up group but not vice versa). The proportion ‘lost to follow-up’ remained consistent at 13.5–16.3% for all the time periods. Most (14/20, 70%) relapses occurred at 6–12 months following treatment, with a mean ±SD time to relapse of 9.6±3.0 months. Relapse rates were 0%, 0.3%, 3.2%, and 1.9% for patients completing 3, 6, 12, and 15 months following treatment respectively. The cumulative probability of relapse following treatment is shown on the Kaplan–Meier survival curve in Figure 3 and was 0%, 1.1%, 2.3%, 3.3% and 4% for patients completing 3, 6, 12, 15 and 18 months respectively.

**Table 5:** Long-term outcomes of visceral leishmaniasis patients treated with 20 mg/kg Ambisome and monitored by active follow-up.

	Follow-up time post-treatment*			
	Completed 3 months	Completed 6 months	Completed 12 months	Completed 15 months
Sample size	984	913	444	196
Lost to follow-up	157 (15.6%)	146 (16.0%)	61 (13.5%)	32 (16.3%)
Death during follow-up period (all-cause mortality)	0	3 (0.1%)	1 (0.9%)	3 (1.2%)
Relapse	0	2	14	4
Relapse rate (95% CI)**	0 (0–0.4)	0.3 (0.04–0.9)	3.7 (2.1–5.9)	2.4 (0.8–5.8)

Time periods and lost to follow-ups are progressive and inclusive, but deaths and number of relapses are mutually exclusive.

Excludes Lost to Follow Up in denominator.

## Discussion

### Patient characteristics

This study cohort represents the largest number of VL patients treated with liposomal amphotericin B (Ambisome) to date worldwide. Although based in one district only, this program has treated an estimated 5.8% of all reported VL cases in India between 2008 and 2011 [2]. Age distributions of patients with VL in the subcontinent context have been described in other epidemiological descriptions in India [11], [12] and Bangladesh [13] which also identified the lower proportion of reported female cases in comparison to the background populations; however, in this study the clear under-representation of older females being treated is of interest and raises the question of whether adult females are less likely to access treatment and have poorer outcomes in this setting. Indeed, this situation has been observed in the Bangladeshi setting where one population based survey among 2,348 people demonstrated a case-fatality rate of 19% among adult women, compared with 6–8% among other demographic groups [14]. However, although health-care facilities in many regions report more male than female cases, the sex ratio can be accurately ascertained only in community-based studies, as data from facilities reflect any disparities in access to health care [15]. Further qualitative research into this phenomenon in the Indian context would be invaluable.

Only 15.5% of the cohort in this program described themselves as being from a forward caste. The remainder was from backward classes or scheduled tribes and castes, supporting the evidence that VL is a disease of the poor and most vulnerable [16]. In India caste can be seen as a proxy for socioeconomic status [17] and is an important determinant of social position; therefore, it affects numerous aspects of daily life [8]. Additionally, members of the same caste tend to live within specific areas, known as ‘*tolas*’, within villages. Together with the poor quality of housing and level of poverty, the well known spatial clustering of VL transmission could contribute to this relationship between low caste and VL. Associations between low caste, reduced access to treatment and more clinically severe VL in Bihar have already been described elsewhere [18], as have the associations between the damp floors, mud plastered walls [19], poor quality thatched housing [20] and high household population density [21] typically seen in lower caste households. As such, in Bihar it is imperative that government schemes such as the Indira Awaas Yojana social welfare programme, designed to provide quality housing for the rural poor in India, be encouraged, implemented and availed.

Children appeared to reach the point-of-treatment earlier than adults in this cohort, and although female sex was a marginal risk factor for late presentation, there was no evidence that in-program mortality was worse for females. A relatively large number of pregnant patients with VL (n = 49) were treated with Ambisome and their outcomes were all good; it is unfortunate that the stage of pregnancy and long-term outcomes of mother and child were not routinely recorded.

A large proportion of patients appeared to be malnourished on presentation to the program. Considering the high background prevalence of malnutrition in Bihar [22], it is difficult to determine the relationship between malnutrition and symptomatic VL in this cohort, and to what degree patients were malnourished prior to being infected by *L. donovani*, as opposed to becoming malnourished as a consequence of VL. There is, however, evidence that malnutrition is associated with early visceralization in *L. donovani* infection and with disease severity [23]–[25]. Providing effective and sustained treatment for the nutritional component of VL proved challenging in this setting because of the short inpatient stays and lack of local nutrition therapies—it is likely to remain so as new shorter-course VL therapies are developed. Particularly alarming is the 18.3% prevalence of severe acute malnutrition among children aged <5 years with VL. These patients are at an increased risk of overall mortality with this degree of wasting [26].

It is well established that HIV-positive patients with VL have poorer outcomes [27]. A previous study conducted within the same center using the same treatment regimen showed increased relapse rates and, in particular, early mortality associated with HIV–VL co-infection [28]. As a result of these outcomes and the numbers of patients being newly-diagnosed with HIV as a result of their VL presentation, MSF started offering all patients voluntary counseling for HIV testing in 2011.

### Treatments

Prior to 2005, the pentavalent antimonial SSG was the widely available first-line treatment for VL recommended by the National Programme in India for more than half a century. However, in addition to the established toxicity of SSG, there is increasing evidence of rising resistance [29], and reports of treatment failure as high as 65% [30]. This has resulted in the gradual introduction of the synthetic phospholipid derivative hexadecylphospocholine, miltefosine (MF) as first-line treatment in VL. MF is a 28-day oral treatment that initially showed promising efficacy and tolerability [31]–[33]. However, its use is restricted in pregnant and lactating women due to its teratogenicity, requiring a minimum of 3 months contraceptive cover during and following the completion of treatment [34]; a recent population pharmacokinetic modeling study of MF has suggested that contraceptive cover should be extended to 5 months [35]. More recent evidence from India has suggested relapse rates of 6.8% at 6- months following treatment with MF [36], which is double that reported in the original study [37]. A separate Indian study demonstrated relapse rates of 7.6% at 12-months following treatment with MF [38]. In the Nepalese context, the relapse rates following MF treatment appear substantially worse with reports of 10.8% and 20.0% at 6 and 12 months, respectively [39]. Measurement of in-vitro susceptibility to MF in patients with relapsed VL has shown lower susceptibility than that found in pre-treatment isolates [40].

Intravenous amphotericin B deoxycholate remains the second-line treatment recommended for VL by the National Programme in India. Although effective, the treatment regimen requires prolonged hospital stays of up to 30 days and has a substantial toxicity profile [15]. Liposomal preparations of amphotericin B allow higher doses to be safely given in a shorter time frame. Over the past decade, numerous studies have been conducted in India examining the effectiveness of liposomal formulations of amphotericin B as monotherapy [7] and in combination [41]. These have shown efficacy ranges >90% for liposomal amphotericin B doses ranging from 5–20 mg/kg. A key phase III study by Sundar et al. demonstrated the safety and >95% efficacy of a single-dose regimen of liposomal amphotericin B 10 mg/kg at 6 months [42]. The study was pivotal in the adoption of this regimen as first-line treatment for VL in South East Asia by the World Health Organization Expert Committee [15]. However, common limitations of these studies include the small cohort sizes and the lack of validation of the results under field conditions.

There are several concerns regarding the introduction of liposomal amphotericin B into national programs, particularly in India. Firstly, the cost of the ‘gold standard’ preparation (Ambisome) is significantly higher than that of other treatment options, despite the agreed company ‘access price’ of USD 18 per 50 mg vial for use in developing countries [29]. However, a cost-effectiveness analysis comparing 10 different treatment modalities showed that if this price was reduced to below USD 9.80 per vial, single dose 10 mg/kg Ambisome would become the most cost-effective treatment [43]. A second concern is the capacity of the healthcare systems of VL endemic countries to maintain the necessary cold chain if Ambisome is to be used in rural areas. Thirdly, because Ambisome is given as an intravenous infusion, it could be considered technically challenging to correctly prepare and administer in those areas where there are limited numbers of qualified nurses and doctors.

The outcomes of this MSF-supported programme provides strong evidence that many of these challenges can be overcome. The successful use of Ambisome to treat 1397 patients in rural PHCs with existing government staff suggests that, with appropriate support and training, it is possible to provide high-quality care for VL patients in such rural settings. A functional cold chain ILR requiring a range of 2–8°C to store vaccines for the Expanded Programme of Immunization in India already exists in the majority of rural PHCs, and vaccination in even the most rural areas is already well established in India. The present program has shown that providing an additional ILR for the storage of Ambisome is a pragmatic solution to the cold chain issue, especially considering the limited number of districts in India where VL is endemic. However, careful monitoring and maintenance of the ILRs remains essential. Increased availability of electronic thermometers to improve the identification of deviations outside set temperature ranges is recommended, as is the inclusion by the manufacturer of a visual vial indicator that will make it easier to identify and discard vials that have been exposed to high temperatures. At the district hospital level ILRs remain an option, but as the required storage temperature of Ambisome ranges between freezing and 25°C, storage in air-conditioned rooms is also a possibility, provided 24-hour generator back-up is available.

### The need for task shifting

This program has also demonstrated that a lack of highly-skilled clinic staff is not a barrier to using ‘complex’ treatments such as Ambisome at the rural PHC level. After appropriate training, lesser-skilled health workers (e.g. dressing nurses) were able to independently prepare, administer, and monitor Ambisome treatment. As suggested by the exceptionally low default rate from ambulatory care, the delegation of responsibility and empowerment (or task shifting) for the management of VL patients to such individuals, following diagnosis by the PHC doctor, also meant that more thorough patient counseling and health education was possible at the point-of-care since the care provider had more opportunity to spend time with patients. This is important when considering the time constraints and heavy patient loads that are a daily reality for the more skilled health workers in the PHCs of India, which is reflected in the generally poor community perception of the quality of PHC care in India [8]. The low mortality rate at the PHC level also suggests that the health workers were able to identify and refer 22.9% of VL patients defined as higher-risk by the MSF protocol, who may have been better served by further assessment and treatment at higher centers of care. However, this conclusion would be better supported by an audit of the appropriateness of the referrals. The present study has also shown that patients accessing care at the PHC level present earlier than those seeking care at the hospital level. Ambulatory treatment at the community level has clear benefits from both societal and healthcare provider perspectives, and is a policy that should be encouraged. However, it is clear that without a change in the community perception of the PHC system in this context, which itself must be based on a good quality of care provision, the full potential of community/rural based management of VL will never be realised.

In conclusion, although expensive, the 20 mg/kg Ambisome regimen is a safe, effective, and feasible treatment for VL patients in Bihar, India. With few severe adverse events, it was well accepted by both patients and medical staff within this MSF-supported program. The active follow-up survey performed after 4-years of routine use indicated that the VL relapse rate remains exceptionally low within 6 months of treatment; however, there is a substantial number of relapses at 6–12 months post-treatment. With a move towards a 10 mg/kg single-dose liposomal amphotericin B regimen and shorter-course combination therapies, and the target of disease elimination in mind, we suggest that 1-year follow-up monitoring be recommended for VL patients in these newer treatment modalities whose longer-term efficacy has yet to be established.

## Supporting Information

Checklist S1 STROBE checklist.
(DOC)

Table S1 Hb values at admission for VL patients by age and sex.
(DOCX)

Table S2 Nutritional status at admission of VL patients by age.
(DOCX)
