# Correction: Five-Year Field Results and Long-Term Effectiveness of 20 mg/kg Liposomal Amphotericin B (Ambisome) for Visceral Leishmaniasis in Bihar, India

**DOI:** 10.1371/journal.pntd.0002812

**Published:** 2014-04-11

**Authors:** The PLOS Neglected Tropical Diseases Staff

The seventh author's name is spelled incorrectly. The correct name is: Manica Balasegaram.
