# Supplementary material for: Five-Year Field Results and Long-Term Effectiveness of 20 mg/kg Liposomal Amphotericin B (Ambisome) for Visceral Leishmaniasis in Bihar, India
Source: PLoS Negl Trop Dis. 2014 Jan 2;8(1):e2603. doi: 10.1371/journal.pntd.0002603 (PMC3879255; doi:10.1371/journal.pntd.0002603)
Supplement: Table S1 — Hb values at admission for VL patients by age and sex. (DOCX) [file pntd.0002603.s002.docx]

| Age group, years | Mean Hb  Male (SD)  (n=4988) | Mean Hb  Female (SD)  (n=3735) | Mean Hb  Overall (SD)  (n=8723) |
| --- | --- | --- | --- |
| <5 (n=598) | 7.0±1.9 | 6.5±1.8 | 6.7±1.8 |
| 5 to <15 (n=3271) | 8.2±1.8 | 7.6±1.8 | 7.9±1.8 |
| 15 to <45(n=3592) | 9.6±2.3 | 7.9±1.9 | 8.9±2.3 |
| ≥45 (n=1262) | 9.1±2.2 | 8.3±1.9 | 8.9±2.1 |
