# Supplementary material for: Five-Year Field Results and Long-Term Effectiveness of 20 mg/kg Liposomal Amphotericin B (Ambisome) for Visceral Leishmaniasis in Bihar, India
Source: PLoS Negl Trop Dis. 2014 Jan 2;8(1):e2603. doi: 10.1371/journal.pntd.0002603 (PMC3879255; doi:10.1371/journal.pntd.0002603)
Supplement: Table S2 — Nutritional status at admission of VL patients by age. (DOCX) [file pntd.0002603.s003.docx]

|  | | | Age group | | | | | | | | Total |
| --- | --- | --- | --- | --- | --- | --- | --- | --- | --- | --- | --- |
|  |  |  | <5 | 5-<15 | 15-<25 | 25-<35 | 35-<45 | 45-<55 | 55-<65 | >65 |  |
| Nutritional  status | Normal | N | 298 | 1294 | 650 | 698 | 601 | 349 | 271 | 131 | 4292 |
|  |  | % | 55.7% | 49.3% | 58.5% | 68.1% | 69.5% | 65.6% | 72.5% | 69.7% | 59.2% |
|  | SAM | N | 98 | 560 | 175 | 162 | 130 | 106 | 48 | 27 | 1306 |
|  |  | % | 18.3% | 21.3% | 15.8% | 15.8% | 15.0% | 19.9% | 12.8% | 14.4% | 18.0% |
|  | MAM | N | 139 | 770 | 286 | 165 | 134 | 77 | 55 | 30 | 1656 |
|  |  | % | 26.0% | 29.3% | 25.7% | 16.1% | 15.5% | 14.5% | 14.7% | 16.0% | 22.8% |
| Total | | N | 535 | 2624 | 1111 | 1025 | 865 | 532 | 374 | 188 | 7254 |
|  |  | % | 100% | 100% | 100% | 100% | 100% | 100% | 100% | 100% | 100% |
